# Biological effects of a semiconductor diode laser on human periodontal ligament fibroblasts

**DOI:** 10.5051/jpis.2010.40.3.105

**Published:** 2010-06-25

**Authors:** Eun-Jeong Choi, Ju-Young Yim, Ki-Tae Koo, Yang-Jo Seol, Yong-Moo Lee, Young Ku, In-Chul Rhyu, Chong-Pyoung Chung, Tae-Il Kim

**Affiliations:** Department of Periodontology and Dental Research Institute, Seoul National University College of Dentistry, Seoul, Korea.

**Keywords:** Alkaline phosphatase, Extracellular signal-regulated kinases, Fibroblasts, Periodontal ligament, Semiconductor diode lasers

## Abstract

**Purpose:**

It has been reported that low-level semiconductor diode lasers could enhance the wound healing process. The periodontal ligament is crucial for maintaining the tooth and surrounding tissues in periodontal wound healing. While low-level semiconductor diode lasers have been used in low-level laser therapy, there have been few reports on their effects on periodontal ligament fibroblasts (PDLFs). We performed this study to investigate the biological effects of semiconductor diode lasers on human PDLFs.

**Methods:**

Human PDLFs were cultured and irradiated with a gallium-aluminum-arsenate (GaAlAs) semiconductor diode laser of which the wavelength was 810 nm. The power output was fixed at 500 mW in the continuous wave mode with various energy fluencies, which were 1.97, 3.94, and 5.91 J/cm^2^. A culture of PDLFs without laser irradiation was regarded as a control. Then, cells were additionally incubated in 72 hours for MTS assay and an alkaline phosphatase (ALPase) activity test. At 48 hours post-laser irradiation, western blot analysis was performed to determine extracellular signal-regulated kinase (ERK) activity. ANOVA was used to assess the significance level of the differences among groups (*P*<0.05).

**Results:**

At all energy fluencies of laser irradiation, PDLFs proliferation gradually increased for 72 hours without any significant differences compared with the control over the entire period taken together. However, an increment of cell proliferation significantly greater than in the control occurred between 24 and 48 hours at laser irradiation settings of 1.97 and 3.94 J/cm^2^ (*P*<0.05). The highest ALPase activity was found at 48 and 72 hours post-laser irradiation with 3.94 J/cm^2^ energy fluency (*P*<0.05). The phosphorylated ERK level was more prominent at 3.94 J/cm^2^ energy fluency than in the control.

**Conclusions:**

The present study demonstrated that the GaAlAs semiconductor diode laser promoted proliferation and differentiation of human PDLFs.

## INTRODUCTION

Lasers have been introduced in the field of periodontology for their adjunctive beneficial effects on conventional periodontal treatments [1-3]. Several researchers have reported that lasers show hemostatic and bactericidal effects, which secures good periodontal treatment results [4-6]. Among various lasers used for periodontal purposes, semiconductor diode lasers are mainly applied in subgingival curettage and periodontal pocket disinfection in clinics [7,8]. They can transform electric energy to light using the same principle as a light-emitting diode, but with internal reflection capability, thus forming a resonator where a stimulated light can reflect back and forth, allowing only a certain wavelength to be emitted Because a semiconductor diode laser has low energy density, this laser is often used for low-level laser therapy, which accelerates the wound healing process without imparting any thermal effect [9-13].

Periodontal wound healing is a series of interactions among periodontal tissue cells, which includes gingival fibroblasts, osetoblasts, cementoblasts, and periodontal ligament fibroblasts (PDLFs). In these cell populations, PDLFs have been regarded to be quintessential for maintaining the periodontium, as it has been suggested that PDLFs could differentiate into osteoblasts and cementoblasts for restoring lost alveolar bone and cementum [14,15].

While low-level laser therapy has been regarded as an adjunctive periodontal treatment modality in clinical therapy, there have been few reports on its effects on PDLFs per se [16,17]. Hence, we investigated the biological effects of low-level lasers on PDLFs by using semiconductor diode lasers in this study.

## MATERIALS AND METHODS

### Cell cultures

Human PDLFs cell lines (ScienCell, San Diego, USA) were maintained in α-MEM (Hyclone, Logan, USA) containing a 10% FBS, and 1% penicillin-streptomycin solution (5,000 units/mL penicillin and 50 µg/mL streptomycin), and these cultures were incubated at 37℃ in a humidified atmosphere in the presence of 5% CO_2_. PDLFs were allowed to attach for 24 hours before being subject to laser irradiation. Prior to the irradiation, the medium was removed and 500 µL of fresh medium was added after the laser irradiation.

### Low-level laser irradiation

**Figure 1:**
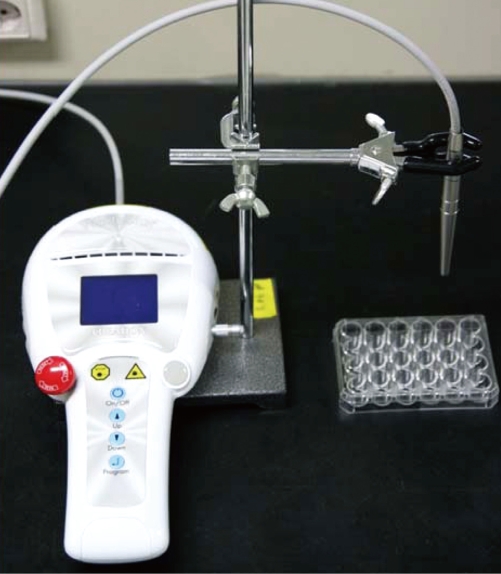
A gallium-aluminum-arsenate semiconductor diode laser with an 810-nm wavelength was prepared and the fiber tip was perpendicularly positioned at a 10-cm distance from the cell monolayer.

An 810-nm gallium-aluminum-arsenate (GaAlAs) semiconductor diode laser (WhiteStar™, Creation, Verona, Italy) was used. Laser light was delivered through a 600 µm fiber-optic system with 500 mW power output in the continuous wave mode. The fiber tip was perpendicularly positioned at a 10-cm distance from the cell monolayer (Fig. 1). Laser beam energy was determined by means of the power meter (FieldMaster™, Coherent Inc., Santa Clara, USA) and irradiation time was 10, 20, or 30 seconds corresponding to energy fluencies of 1.97, 3.94, and 5.91 J/cm^2^, respectively. The control cultures were treated equally, except for the laser irradiation.

### Cell proliferation assay

A 3-(4,5-dimethylthiazol-2-yl)-5-(3-carboxymethoxyphenyl)-2-(4-sulfophenyl)-2H-tetrazolium (MTS) assay was performed to assess the cell proliferation activity. PDLFs were plated at a density of 1 × 10^4^ cells/well in a 24-well culture plate and after laser irradiation all groups were incubated for 24, 48, and 72 hours. Then the cell medium was removed and replaced by fresh medium containing MTS reagents (Promega, Madison, USA). After 3 hours of incubation, the absorbance at 490 nm was measured using a spectrophotometer (Molecular Devices, Sunnyvale, USA).

### Alkaline phosphatase activity test

PDLFs were plated at a density of 1 × 10^5^ cells/well in a 24-well culture plate and all groups were incubated for 24, 48, and 72 hours after the laser irradiation. PDLFs were washed three times with phosphate buffered saline (PBS) and solubilized with alkaline buffer. The lysates with the alkaline phosphatase (ALPase) assay working solution were incubated for 4 minutes. After incubation, the absorbance of p-nitrophenol was read at 405 nm using a microplate reader (Molecular Devices, Sunnyvale, USA). The total cell protein was measured using a protein assay kit (Invitrogen, Carlsbad, USA) and the results were expressed in µmol/60 minutes/µg.

### Western blot analysis for extracellular signal-regulated kinase (ERK) activation

Laser-irradiated PDLFs were plated at a density of 1 × 10^5^ cells/well in a 24-well culture plate and incubated for 48 hours, after which they were washed with PBS. The cells were then lysed with RIPA buffer. The protein lysates were centrifuged at 13,000 g for 30 minutes, and the supernatant was collected for protein quantification using the protein assay kit (Invitrogen, Carlsbad, USA). Total protein in the amount of 50 µg was boiled and separated electrophoretically on SDS-PAGE gel and transferred onto a Hybond-P polyvinylidene difluoride (PVDF) membrane (GE Healthcare, Piscataway, USA) by electroblotting. After blocking in TBST buffer (25 mM Tris-HCl pH 7.4, 1.5 M NaCl, 0.5% Tween-20) containing 5% fat-free dry milk for 1 hour, the PVDF membrane sheets were incubated overnight at 4℃ with primary antibodies. The primary antibodies used were anti-phosphor-ERK (Cell Signaling, Beverly, USA), anti-β-actin (Santa Cruz Biotechnology, Santa Cruz, USA). After washing three times with TBST, the membrane sheets were incubated with horseradish-peroxidase conjugated secondary anti-rabbit antibody (Sigma, St. Louis, USA) for 1 hour. The immunoreactive bands were visualized using a luminescent image analysis system (Fujifilm Life Science, Waipahu, USA).

### Statistical analysis

The statistical analysis was performed by a statistical software package (SPSS™, SPSS Inc., Chicago, USA). The mean values and the standard deviation were calculated for the MTS assay. The data were analyzed by ANOVA with an adhoc Tukey's test to assess the significance level of the differences among the energy doses. The statistical significance was set at *P*<0.05.

## RESULTS

### Cell proliferation assay

**Figure 2:**
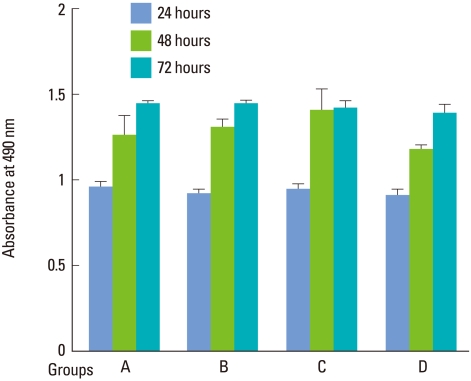
Human periodontal ligament fibroblast proliferation after 810-nm semiconductor diode laser irradiation at the energy fluencies of 1.97 (B), 3.94 (C) and 5.91 (D) J/cm^2^. Periodontal ligament fibroblast proliferation was gradually increased up to 72 hours without any significant difference compared with the control (A) at all levels of irradiation.

**Figure 3:**
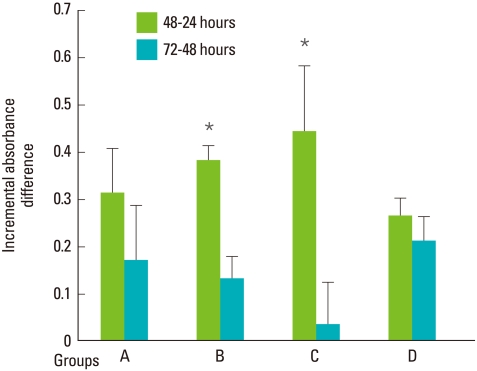
Incremental difference in absorbance at 490 nm in human periodontal ligament fibroblasts (PDLFs). PDLFs proliferation between 24 and 48 hours was significant at the energy fluencies of 1.97 (B) and 3.94 (C) J/cm^2^, but was not in the 5.91 (D) J/cm^2^ and control group (A) (^*^P<0.05).

After GaAlAs semiconductor diode laser irradiation of human PDLFs for 10, 20, or 30 seconds, increased cell proliferation was recorded in a time-dependent manner. At all levels of applied irradiation, human PDLFs proliferation gradually increased for 72 hours (Fig. 2). While there was no significant difference compared with the control over the entire 72 hours taken together, significant incremental PDLFs proliferation was observed between 24 and 48 hours at both 1.97 and 3.94 J/cm^2^ energy fluencies (Fig. 3).

### ALPase activity test

**Figure 4:**
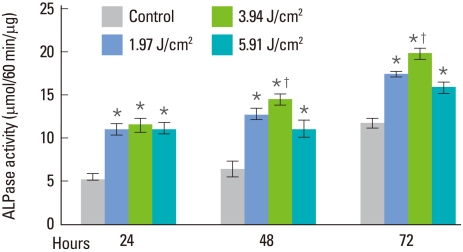
Effect of semiconductor diode laser irradiation on alkaline phosphatase (ALPase) activity in human periodontal ligament fibroblasts (PDLFs). All the laser-irradiated groups showed a significant increase compared to the control. Among the laser-irradiated groups, ALPase activity of PDLFs was significantly greater at 3.94 J/cm^2^ of laser energy fluency. ^*^Statistically significantly greater than the control (*P*<0.05). ^†^Statistically significantly greater than other laser-irradiated groups (*P*<0.05).

The effect of laser irradiation on the ALPase activity of human PDLFs is illustrated in Fig. 4. Compared with the control, significantly increased ALPase activity of laser-irradiated PDLFs was revealed regardless of the amount of laser energy fluency or irradiation time (*P*<0.05). Among the irradiated groups, the irradiated PDLFs group with 3.94 J/cm^2^ of laser energy fluency-showed statistically significant ALPase activity at 48 and 72 hours (*P*<0.05).

### Western blot analysis

**Figure 5:**
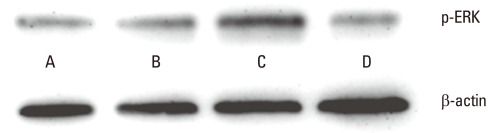
Result of western blot analysis of phosphorylated extracellular signal-regulated kinase (ERK) at the laser energy fluencies of 1.97 (B), 3.94 (C) and 5.91 (D) J/cm^2^ compared to the control (A). 3.94 J/cm^2^ of laser energy-fluency promoted significantly greater ERK activation.

Western blot analysis showed that phosphorylated ERK activity was prominent in the laser-irradiated PDLFs group at 3.94 J/cm^2^ (Fig. 5).

## DISCUSSION

It has been reported that a low-level laser can accelerate wound healing [10-13], enhance bone and collagen formation [18-23] and induce anti-inflammatory effects [18-20]. These findings are supported by *in vitro* examinations confirming that low-level laser irradiation significantly increases cell proliferation [16,21] and collagen deposition [22], and enhances osteogenic differentiation [23].

Studies aiming to confirm the effects of low-level lasers on PDLFs, however, are very few. The responsiveness of PDLFs to low-level laser energy *in vitro* has been demonstrated by Shimizu et al., who showed that 830 nm GaAlAs laser irradiation of stretched human periodontal ligament cells significantly inhibited the production of prostaglandin E_2_ and IL-1β [24]. Furthermore, there have seldom been reports regarding the proliferation and differentiation of PDLFs in general.

In the present study, we revealed that an 810 nm GaAlAs semiconductor diode laser increased human PDLFs proliferation, although there were no significant differences among groups. The present result is inconsistent with that of Kreisler et al. [16], who discovered that laser irradiation stimulated the proliferation of PDLFs. In their study, a GaAlAs diode laser with a wavelength of 809 nm significantly increased the metabolic activity of PDLFs with a duration of incubation of up to three days. They also reported that laser energy fluencies of 1.96, 3.92, and 7.84 J/cm^2^ had similar effects on PDLFs. Our current findings are different from theirs. PDLFs proliferation increased in time-dependant manner up to 72 hours irrespective of irradiation regimen. The reasons for the inconsistency could lie in the differences in power output, fiber distance from the cell monolayer, and irradiation time.

The level of ALPase of periodontal ligament cells is known to be one of the initial differentiation markers of osteoblasts because this level increases in the initial differentiation stage [25]. Hence, we investigated ALPase activity in order to evaluate the differentiation of PDLFs after laser irradiation and found that low-level semiconductor diode laser irradiation significantly stimulated PDLFs differentiation at all energy fluencies of 1.97, 3.94, and 5.91 J/cm^2^. Among the groups, the 3.94 J/cm^2^ lased group showed significantly greater results compared to the other irradiation groups. This suggests that low-level laser irradiation with a 3.94 J/cm^2^ energy fluency is suitable for PDLFs differentiation.

The present study found, first, that low-level laser irradiation activated ERK. In general, the mitogen-activated protein kinase (MAPK) pathway regulates cellular responses to environmental changes [26]. ERK, which is a typical MAPK family member, is activated by several growth factors and mitogens to induce cell proliferation and differentiation [27]. We revealed that 3.94 J/cm^2^ energy fluency more prominently activated ERK of human PDLFs than other energy fluencies and the control. These findings coincide with a previous report which revealed that laser irradiation at 3 and 4 J/cm^2^ exerted stimulatory effects, whereas 5 J/cm^2^ caused inhibitory effects in NIH-3T3 fibroblasts [22].

Interestingly, the incremental PDLFs proliferation between 24 and 48 hours revealed significantly greater values at 1.97 and 3.94 J/cm^2^ energy fluencies. In a previous report, the difference in PDLFs proliferation on days 1 and 2 after laser irradiation was highly significant and decreased on day 3 [16]. Our data regarding the incremental cell proliferation is comparable to those of previous studies, which showed peaked molecular and cellular responses up to 2 days after laser irradiation [16,28]. We can deduct that low-level laser irradiation affects the cellular environment in the early incubation period within 2 days, which requires further investigation.

In conclusion, we demonstrated that the GaAlAs semiconductor diode laser could stimulate proliferation and differentiation of human PDLFs. Within the limits of this study, the optimal energy fluency for the stimulation of PDLFs differentiation was 3.94 J/cm^2^.
